# Contextualizing the experiences of Black pregnant women during the COVID-19 pandemic: ‘It’s been a lonely ride’

**DOI:** 10.1186/s12978-023-01670-4

**Published:** 2023-08-25

**Authors:** Alicia A. Dahl, Farida N. Yada, Shanika Jerger Butts, Annalise Tolley, Sophie Hirsch, Priyanka Lalgondar, Kala S. Wilson, Lindsay Shade

**Affiliations:** 1 Department of Public Health Sciences, University of North Carolina at Charlotte, 9201 University City Blvd, Charlotte, NC 28223 USA; 2 Department of Psychological Sciences, University of North Carolina at Charlotte, 9201 University City Blvd, Charlotte, NC 28223 USA; 3 Department of Family Medicine, Atrium Health, 2001 Vail Avenue, Suite 400-B Mercy Medical Plaza, Charlotte, NC 28207 USA

**Keywords:** Black or African American, Maternal health, Pregnancy, COVID-19, Qualitative research, Social adjustment, Mixed methods

## Abstract

The emergence of the COVID-19 pandemic significantly changed the prenatal care experience, specifically regarding medical appointments and social opportunities. It is critical to capture this change through the narratives of pregnant people, particularly those of marginalized populations, whose voices may often be underrepresented in the literature. This mixed-methods paper summarizes the experiences of 40 pregnant Black/African American (AA) women during the COVID-19 pandemic. A cross-sectional, online survey was administered between 2020 and 2021 to assess prenatal health and the impacts of the COVID-19 pandemic on patients’ pregnancy experience. Coping behaviors during the pandemic were self-reported using the COPE-IS. Univariate analyses were conducted. An additional analysis of participants (*n* = 4) was explored through a week-long qualitative exercise using a photo documentation procedure. Photo-Elicitation Interviews (PEI) were conducted to capture and center their pandemic pregnancy experiences. Sources of stress during the pandemic varied, with the most common being financial concerns (*n* = 19, 47.5%). Over half of the sample (*n* = 18, 54.5%) self-reported increases in their positive coping behaviors during the pandemic, such as communicating with friends and family, talking to healthcare providers, listening to music, and engaging in spiritual practices–such as prayer. The four PEI study participants reflected on the impacts of social distancing on their prenatal experience and mentioned hospital and provider-related weariness due to their race. The findings of this study suggest that during the COVID-19 pandemic, Black/AA pregnant women in Charlotte, NC used social support, mindfulness practices, self-advocacy, and health literacy to navigate challenges present during their prenatal health experience. This paper highlights the personal, social, and structural experiences of pregnant women during a public health crisis so that responsive and effective programs or policies can be planned in the future.

## Introduction

For a high-income country, the United States (U.S.) is one of the most dangerous countries to give birth [1]. Recent attention has been focused on the increasing national maternal mortality rate (MMR). Between 2018 and 2019, the MMR increased from 17.4 to 20.1 deaths per 100,000 live births nationwide, and over the COVID-19 pandemic years of 2020 to 2021, rates staggered to 23.8 and 32.9, respectively [2]. The MMR for the state of North Carolina (NC) consistently exceeded the national rate from 2019 to 2021, with a doubling effect over the course of the pandemic (22 to 44 deaths per live births, respectively) [3].

Further, MMR data indicate significantly wide racial and ethnic disparities, which the COVID-19 pandemic has exacerbated. From 2019 to 2021, maternal deaths among non-Hispanic Black women rose from 44.0 to 69.9 per 100,000 live births, which is considerably higher than the mortality rate faced by non-Hispanic White women during the pandemic (17.9 to 26.6) [2]. MMR data indicate a similar upward trend among Hispanic women, with 12.6 to 28.0 [2]. These data occur in the context of considerable, multisystemic hardships affecting marginalized communities, which were further amplified by disproportionate impacts of the COVID-19 pandemic across financial (e.g., access to prenatal care), physical (e.g., illness), emotional (e.g., social support), and socio-cultural spheres (e.g., racism), prompting significant concerns for women of color in the U.S., including Black/African American (AA) women experiencing pregnancy [4].

The COVID-19 pandemic changed the collective pregnancy experience, which contributed to a rise in perinatal depression during the pandemic. While mental health concerns increased significantly across the population [5], pregnant women experienced unique stressors, such as managing the anxiety associated with the unknown effects of COVID-19 on the baby, determining whether it was safe to receive the vaccine when available, attending visits and delivering with visitor restrictions, and receiving care via telehealth [6]. In addition, once the baby was born, concerns and barriers may have continued to accumulate, such as inconsistent access to childcare or fear of returning to the workforce in high-risk job settings. Reasons such as these contribute to global rates of pregnant and postpartum depression, which were significantly higher during the pandemic than before, particularly for low-risk pregnancies (i.e., no active complications) [7, 8]. This rise in perinatal depression is a concern within itself. Still, it is also a concern for other maternal and infant health outcomes (e.g., preeclampsia, low birth weight), which can be negatively impacted by perinatal stress [9]. While the unique challenges of pregnancy during the COVID-19 pandemic affected all women [10], it may have had a more significant adverse effect on Black/AA pregnant women, who reported higher rates of COVID-19-related distress [4].

### Structural barriers to Black/AA maternal health

It is essential to acknowledge the historical context of health disparities constructed through structural violence, especially as it relates to Black/AA maternal health [11]. Systemic inequities in healthcare include experimentation on Black/AA bodies [12], unequal access to high-quality care [13], segregation of medical facilities [14], and exclusion of Black/AA individuals from medical education [12]. This systemic disadvantage is baked into healthcare systems today, influencing the quality of care that Black/AA women receive from their providers.

Systemic inequities in the healthcare system are well documented in research. For instance, Wheatley et al. asked women about their perinatal experiences: Black/AA individuals were likelier than their White counterparts to discuss or mention adverse perinatal experiences with their providers [15]. In many cases, Black/AA women perceived their care was of lower quality. An additional barrier that Black/AA women face during their pregnancy is a lack of same-race and culturally humble providers (i.e., providing care that reflects the cultural context of the patient) [16], which may negatively influence communication, trust, and satisfaction [17].

### Coping with mental health challenges

With the COVID-19 pandemic, mental health issues significantly increased across populations, including pregnant women [18]. Reducing perinatal psychological distress such as anxiety, depression, and stress is an important goal during every pregnancy since poor mental health throughout the pregnancy experience increases the risk for postpartum depression [19]. There are limited studies that discuss coping strategies and enabling factors that promote health in the face of barriers for this population. Regarding the measurement of coping, Ruiz et al. (2015) addressed the need to test the psychometric properties of instruments for use in minority populations, acknowledging the different experiences and multifactorial nature of pregnancy health outcomes [20].

One enabling factor of perinatal health that may be valuable to Black/AA women is social support. Social support is defined by how social relationships fill the gaps between emotional, affectionate, and tangible needs [21]. Social support may provide a buffering effect during stressful events. The degree of social support provided during pregnancy has been shown to reduce anxiety and other depressive symptoms that lead to self-harm during the perinatal period and postpartum depression [21]. One example is having a significant other present while attending doctor’s appointments, spending time with friends and family, or simply enjoying a meal together. Importantly, these experiences are multifaceted and inconsistent across a population demographic due to varying social determinants of health. For example, there is evidence that low levels of social support can lead to depression in the third trimester for low-income Black/AA pregnant women [20]. The magnitude of social support specifically for Black/AA maternal health outcomes is underrepresented in the broader literature. Research efforts to understand such experiences during the COVID-19 pandemic increased but yielded smaller sample sizes for Black/AA populations [22].

Racial differences exist in the perception of social support and its perceived effectiveness in decreasing depressive symptoms [23–25]. Understanding these differences is important because while perinatal depression is common, Black/AA women are especially at risk compared to White, non-Hispanic women [19]. This risk can be magnified if Black/AA individuals have intersecting identities associated with barriers to their perinatal health.

Other coping responses are noted in the literature. Wheeler et al. found that many pregnant women used faith-based practices (i.e., spirituality and religiosity) to reduce COVID-19-related anxiety [26]. Using spiritual coping during the prenatal period may be particularly important to Black/AA women. In one study, Black/AA women reported being more inclined to accept mental health support through spiritual counseling, and they were more likely to trust a religious leader than a counselor or antidepressants [27]. This may suggest the importance of the cultural and community context when understanding the relevance, salience, and/or acceptability of support mechanisms.

While the literature suggests various approaches to improve maternal health labor and birth outcomes in pregnant women [28–30], it is not yet documented how Black/AA women in Charlotte, NC, cope with distress during their perinatal journey or how these behavioral frequencies have been affected by the COVID-19 pandemic.

### Study aim

This project initially aimed to understand the barriers and enabling factors present in the pregnancy experience of Black/AA women in Charlotte, NC, to inform new public health programs and resources tailored to community needs. A month into study recruitment, the COVID-19 pandemic was declared a global public health crisis, and our research aims shifted to learn about the personal, social, and structural experiences of Black/AA birthing people within the context of the pandemic.

Despite the profound disparities in perinatal experiences that put Black/AA women at greater risk for morbidity and mortality, Black/AA perinatal narratives are absent in the COVID-19 literature. Before the pandemic, multiple sources cited the importance and value of understanding the pregnancy experience and stress-related exposures of Black/AA women to respond effectively to the widening health disparities compared to other racial and ethnic groups [31, 32]. Centering the narratives of pregnant women who have been historically pushed to the margins is critical to ensure those experiences are understood and accounted for in medical practice. Thus, this research relied on standpoint feminist theory [33] and intersectionality theory [34] to shift science away from a privileged male perspective and/or White women’s experience, respectively, while considering participants’ perceived gendered racism. This study aimed to address this gap by contextualizing the experiences of Black/AA pregnant women in their local setting during the COVID-19 pandemic and their coping behaviors using a mixed-methods approach.

## Materials and methods

This study explored the following broad research questions within the context of the COVID-19 pandemic: (1) What makes it challenging for Black/AA women to have a healthy pregnancy (barriers), and (2) What helps Black/AA women to have a healthy pregnancy (support)? The study questions were addressed using mixed methods: (1) participants completed a one-time, cross-sectional questionnaire including a series of domains attempting to quantify their experiences related to stress, healthcare access, social support, and other social determinants of health, and (2) a qualitative participatory exercise was conducted to contextualize the prenatal experiences of Black/AA women during the pandemic using qualitative Photo-Elicitation Interview (PEI) methods. This paper will present findings on sources of stress, coping, and support for Black/AA pregnant women living in Charlotte, NC, during the COVID-19 pandemic.

The PEI method is a strategy used to identify and communicate the needs of a population by tasking community members to photograph aspects of their daily life and subsequently participate in a one-on-one interview and exploratory conversation about their documented images [35]. The PEI method used in this study was selected to empower Black/AA women to share and express their pregnancy experience, to allow them to expand their responses beyond the quantitative options of the survey tool, and to build a shared understanding between participants and the research team. Perceptions of barriers and enabling factors for healthy pregnancy were elicited from the viewpoint of expectant mothers through the photographs [35]. To our knowledge, PEI had not been previously used to understand the prenatal health experiences among Black/AA women in the Charlotte metropolitan area.

The qualitative interviews were conducted to deepen our understanding of how participants navigated social and cultural support for their health and well-being during a public health crisis, which could not be fully captured through the quantitative coping measures. Using a convergent mixed-methods approach with a participatory framework, data were collected in parallel and merged during data analysis [36]. For questionnaire items that indicated common sources of stress and coping practices, similar themes and code applications of the interviews were reviewed. We present the results in a contiguous approach due to the small sample size for the qualitative portion to avoid generalizing the experiences of all study participants. We aimed to center the narrative around the experiences of the Black/AA pregnant women in response to current best practices for conducting research with marginalized populations.

### Researcher positionality

The lead investigator for this project identifies as a White woman with a research focus on community health and perinatal health disparities. The core team of doctoral student trainees involved in this research identifies as Black women, one of whom is a mother. This work was supported by a healthcare provider, who identifies as a White woman and a mother, and an interdisciplinary team of student writers with various identities, experiences, and research concentrations. Racial concordance during the interview process and codebook application for the qualitative portion of the study may improve communication, comfort, cultural humility, and rapport building [37] as documented in the healthcare experience. Findings were reviewed and discussed as a collective process between the research team members (FY, SB, & AD) to ensure the cultural context of experiences shared by participants was retained and honored in the final narrative. The collective interest in public health, the health care system, and improving the health disparities of marginalized groups informed their engagement and perspectives in this project.

### Participant recruitment

To be eligible for participation, women had to be at least 18 years of age, a resident of Mecklenburg County, NC, for at least 1 year, pregnant during recruitment and had to self-identify as Black/AA. The following populations were excluded from participation in this study: men, women younger than the age of 18, non-Mecklenburg County residents or Mecklenburg County residents for less than 1 year, those who did not identify as Black/AA, and those who were not pregnant at the time of recruitment.

Recruitment began in February 2020 and was completed in May 2021. Participants were recruited through five local OB/GYN clinics, university listserv announcements, printed fliers in clinical waiting and exam rooms, and by word-of-mouth. All interested patients completed an online screening questionnaire to determine study eligibility. In-person recruitment occurred at monthly obstetric intake sessions, where a doctor or nurse at the clinic introduced the study to eligible patients. If patients expressed interest, a study team member was invited into the exam room for active, in-person enrollment, consent, and survey participation. Another mode of contact included patients receiving a small card from their provider with the eligibility criteria outlined and a QR code to scan and contact the study team if interested, which was a helpful navigation strategy for recruitment during the height of the pandemic lockdown. There was a 9-month pause for in-person recruitment from mid-March through December 2020 due to restrictions set by local public health authorities and health systems in response to the COVID-19 pandemic, which slowed the enrollment of participants immensely. During this time, online recruitment and passive recruitment strategies were maintained.

For non-clinic recruitment, a research team member followed up with a phone call to eligible candidates to review the study details and answer any questions before confirming enrollment. A maximum of five contact attempts were made with the potential participant using the email or phone number provided. Once eligibility was confirmed and all questions were answered, participants received a link to a Qualtrics questionnaire, where they were prompted to complete the informed consent process before moving through a battery of measures. Upon completion of the survey, participants were asked if they were interested in participating in the PEI segment of the study, and if so, were contacted via email by a study team member with an invitation to attend a virtual orientation session on the next steps.

### Procedures

The research team did not use protected health information or medical chart abstraction for the dataset but instead relied on self-reported data.

### Quantitative measures

The questionnaire took approximately 20–30 min, and participants received a $20 Amazon.com gift card for completion.

#### Demographic characteristics

Eleven items were asked at the start of the survey to capture participant demographics. Items from the demographics section included gestational age (weeks), pregnancy intention (planned/unplanned), parity (prior pregnancies and live births), relationship status, age, educational attainment, employment status, residential zip code, and total household income before taxes. General health status was assessed via eight items from the Pregnancy Risk Assessment Monitoring System [38]. Items asked about self-perception of physical health, height, weight, health care visits, family planning, and preexisting conditions.

#### Sources of stress

Participants were also asked to identify their three greatest sources of stress from the COVID-19 pandemic using a brief and non-exhaustive checklist of 12 responses adapted from the Coronavirus Perinatal Experiences-Impact Survey (COPE-IS) [39]. Response options included health concerns, financial concerns, access to various resources, social distancing, and the impact of the pandemic on their social networks.

#### Coping mechanisms

Participants responded to 16 questions regarding stress-related coping mechanisms used during the COVID-19 pandemic, adapted from COPE-IS [39]. The overall question of the set was, “How have the following coping behaviors changed for you during the COVID-19 pandemic?” The response options were increased, decreased, or no change and included a list of positive (e.g., meditation and mindfulness practices) and negative (e.g., tobacco use) health behaviors for coping. Other response prompts included physical health such as sleep, brain exercises, nutrition, illicit drug use, alcohol consumption, marijuana use, and sedentary and physical activity habits. Broader prompts assessed the social dimension of health, such as how friend and family interactions, healthcare provider communication, volunteer work, and faith-based practices had been impacted due to the COVID-19 pandemic.

### Quantitative statistical analysis

The questionnaire data were cleaned and coded by research team members (AD, FY, SB). As the primary aim was to characterize our specific sample in their unique context, the analysis focused on summarization and description rather than prediction or explanation. Means and standard deviations were calculated as appropriate (e.g., gestational age). Frequency distributions were used to summarize the remaining demographic characteristics of the participants, and no association analysis was conducted, given the limited power of the small sample. All descriptive statistics were prepared using SAS® 9.4 [40].

### Photo-elicitation procedures

We applied Sampson-Cordle’s (2001) approach of having study subjects photograph their realities, followed by an interview process where participants analyze their photographs to develop a narrative [41]. Interested participants attended a virtual orientation session to discuss the goals and objectives of PEI, as well as best practices for collecting photos and ethics (e.g., obtaining consent for pictures of people’s faces). The session also introduced the participants to prompts that would be considered during the week of the photo collection. For example, *“What makes it challenging for Black/AA women to have a healthy pregnancy (barriers)?”* and *“What helps Black/AA women to have a healthy pregnancy (support)?”.*

When participants consented to join the photo-elicitation phase, a study team member invited them to begin photo documentation the next day. Daily prompts were emailed or texted to the participants to encourage photo documentation and a reminder to submit photos to the study team through email. Participation adherence was defined by documenting pictures on 5 of the requested seven days and submitting photos within 2 weeks from enrollment. Implementing this adherence period helped to ensure that participants documented enough photos to provide sufficient material for the interview process. Participants who met this adherence threshold were contacted to schedule an individual interview session with a study team member. Interviews were held over Zoom (San Jose, CA: Zoom Video Communications Inc) and audio recorded. Researchers organized the individual’s collected photos into a slide deck. Using the screen share function, the interviewer led the participant through a semi-structured interview process. The interviewer would re-share the prompts and ask participants to reflect on the submitted photos. The SHOWED method of questioning consists of the interviewer asking the participant to describe what is seen, what is happening in the photographs, how what is pictured relates to their community, why this exists, and what can be done about it [42]. This pattern of questions was repeated for each cluster of photos. Interviews lasted approximately 60–90 min. Participants were eligible to receive up to $80 in Amazon.com gift cards for participating in the PEI process.

### Qualitative analysis

Interview recordings were transcribed via Temi.com. Two graduate student research team members (FY and SB) used a combined approach of creating a codebook from the interview guide (e.g., challenges and supports), followed by a hybrid approach of inductive and deductive thematic analyses upon review of the transcripts [43]. First, we used our PEI prompts and semi-structured interview guide to categorize how participants observed barriers and enabling factors to pregnancy within their lived environment to establish main themes and subthemes (e.g., Challenges, Resources, Barriers, Support). Based on our observations of the photos and captions submitted, an initial codebook was developed by AD, FY, and SJ. Next, FY and SJ independently coded the interview transcripts using an inductive approach, discussing and defining new codes or themes during study team meetings. Finally, a series of meetings were held with the principal investigator (AD) to resolve major discrepancies for codes with a kappa < 0.75 and develop additional code considerations using exemplars as needed. As the lead interviewer, FY provided context regarding the participants’ key points as needed, and interview recordings were made available for review. For example, when a participant described providing advocacy support to other pregnant people in the community, our team revisited whether this belonged as a strength (e.g., social connectedness) or challenge (e.g., burdensome task). Researchers reflected on their positionality during the coding process. All qualitative analyses were conducted in Dedoose Version 9.0.46 [44], a web application for managing, analyzing, and presenting qualitative and mixed methods research data.

Due to the ongoing COVID-19 pandemic at the time of data collection and the inclusion of a high-risk population group (i.e., pregnant people), saturation was reached quickly due to societal shifts in work and leisure behaviors leading to very similar narratives with how the participants were social distancing, working from home, and navigating OBGYN care with restrictions in place. A systematic review of qualitative studies by Hennink and Kaiser concluded that studies could reach saturation with a small sample when homogenous with narrowly defined objectives [45].

## Results

### Quantitative findings

#### Participant demographics

A total of 58 participants were eligible and invited to participate, with 40 participants completing the survey (response rate = 69%). The median age of women in this study was 30 years (SD = 6.15), with the sample ranging from 19 to 41 years old. All of the participants had at least earned a high school diploma, with the majority of the participants earning some level of a college education; associate degree (n = 5, 12.5%), bachelor’s degree (n = 14, 35%), and advanced graduate degree (n = 7, 17.5%). Half of the sample had a combined household income of $50,000 or less (n = 21, 52.5%) compared to a fifth with a reported household income of $100,000 or more (n = 8, 20%). When asked about their work for wages during the COVID-19 pandemic, over half of the sample reported a role that was considered an “essential worker” (e.g., grocery store employee; n = 21, 52.5%). Most of the women in the study were in a partnered relationship of some type (n = 34, 85%), with only 12.5% responding as “single” (n = 5).

Self-perceptions of general health varied among this sample. Generally, ratings were positive, with 15% of the sample selecting “Excellent” (n = 6), 35% rating “Very good” (n = 14), and 32.5% choosing “Good” (n = 13). Six participants rated their overall health as “Fair” (15%), and only one participant selected “Poor” (2.5%). Regarding their prenatal health status, almost half of the participants did not plan to become pregnant at conception (n = 18, 45%), and fifteen participants in the sample were considered first-time moms (37.5%). On average, participants were 20 weeks pregnant (SD = 9.4) at the time of participation. This was considered the second pregnancy for most women in the study (n = 15, 37.5%). Five participants reported a prior pregnancy experience that did not result in a live birth (12.5%). At the time of participation in the survey, only two participants reported ever receiving a COVID-19 diagnosis (5%).

#### Sources of stress during the COVID-19 pandemic

**Fig. 1:**
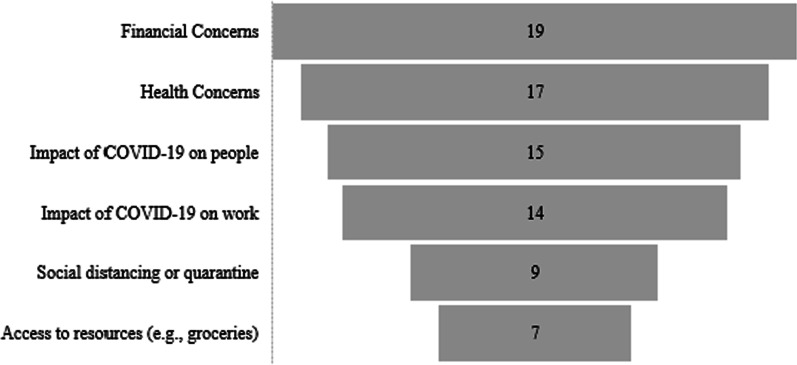
Descriptive statistics of the greatest sources of stress reported by pregnant women during the pandemic

Participants were asked to select their top three sources of stress from a list of 12 items broadly representing the six domains presented in Fig. 1. The most commonly cited sources of stress were financial concerns (n = 19, 47.5%), health concerns (n = 17, 42.5%), and the impact of COVID-19 on people in their personal networks (e.g., children, family, community) (n = 15, 37.5%) (Fig. 1).

#### Coping mechanisms

Thirty-three participants responded to questions about behavior changes around meal preparation, physical exercise, and time spent in nature during the COVID-19 pandemic, with only two participants indicating no changes (6.1%). Over half of the respondents noted eating more home-cooked meals (n = 18, 54.5%), and 39.4% described eating more takeout/delivered food (n = 13). Regarding physical exercise, nearly half of the respondents got less exercise than usual due to the pandemic (n = 15, 45%), while only 9% saw an increase in exercise behaviors (n = 3). Lastly, time spent in nature during the COVID-19 pandemic decreased for most participants (n = 17, 51.5%), while a fifth of the sample reported increased exposure to nature (n = 7, 21.2%). The frequency of snacking increased for nearly 70% of the respondents during this period (n = 23).

**Fig. 2:**
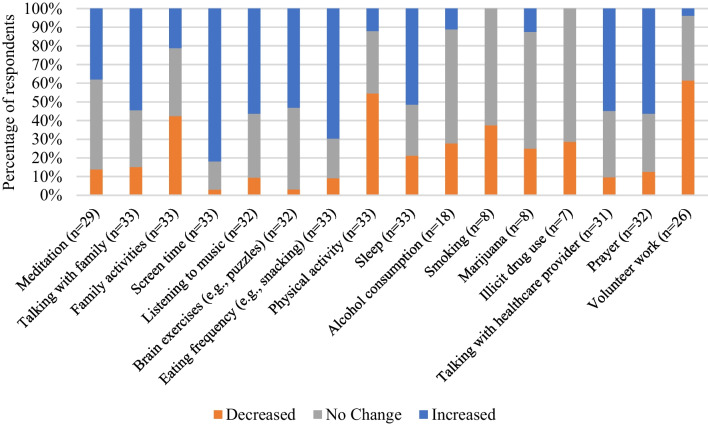
Descriptive statistics of applied participant strategies used to cope with stressors during the COVID-19 pandemic

When surveyed about behaviors or strategies used for coping with stress during the COVID-19 pandemic, 33 participants responded. A list of 16 mechanisms for coping was provided with a “select all that apply” prompt. For the selected behaviors, participants indicated whether there was an observable increase, decrease, or no change. As shown in Fig. 2, most of the respondents (n = 27, 81.8%) reported increased screen time (e.g., watching tv, video games, and smartphone use) as a coping mechanism. Another highly cited change in coping behaviors was around eating (e.g., snacking), with 23 participants reporting an increase (69.7%). Eighteen women noted a decrease in physical activity for coping (54.5%) compared to four women who reported an increase (12.1%). Over half of the respondents (n = 18, 54.5%) reported a rise in behavior for music listening, talking with family, or engaging in prayer or faith-based practices. Slightly over half of the women reported an increase in sleep (n = 17, 51.5%), while seven (21.2%) reported decreased sleep. Additionally, participants reported a decrease in their consumption of alcohol (n = 5, 15.2%), tobacco use (n = 3, 9.1%), marijuana use (n = 2, 6%), and other illicit drug use (n = 2, 6%), which may be reflective of typical pregnancy-related health behavior change and not the context of coping mechanisms during the pandemic.

Social support was explored with items about how talking with family and friends changed during this time. Over half of the women reported increased communication with family and friends (n = 18, 54.5%). In comparison, 42.4% (n = 14) reported no change in the communication patterns with their family and friends, and 15.2% (n = 5) indicated decreased communication. Less than a quarter of women (n = 7, 21.2%) reported increasing the amount of time spent participating in family activities (e.g., games, sports), and 42.4% responded that these types of family interactions had decreased during the pandemic (n = 14). Just over half of the participants reported an increase in talking with their healthcare providers (e.g., medical doctor, mental health provider) (n = 17, 51.5%), and a third of women reported no change (n = 11, 33.3%) regarding how often they spoke with their healthcare provider.

When asked how their faith-based practices had changed during the COVID-19 pandemic, over half of the women (n = 18, 54.5%) reported that these practices had increased. On the contrary, 12.1% (n = 4) reported a decrease in their frequency of faith-based practices, while a third indicated no change (n = 11, 33.3%). Engagement in meditation practices increased for 33.3% of women (n = 11) and decreased for 12.1% (n = 4), while no change in meditation practice was reported by nearly half of the women (n = 14, 42.4%).

### Photo-elicitation interview findings

#### Participant characteristics

Six of the 40 survey participants attended the PEI orientation sessions (15%), with four total respondents agreeing to participate in the PEI. The four participants were between the ages of 31–40 years. Three participants were married, while one was in a relationship. At the time of the study, three participants were enrolled in school, and one was self-employed. All of the participants were educated beyond high school, including some college (n = 1), college (n = 2), and an advanced degree (n = 1).

#### Photo summary

The four participants shared 122 photos in response to our text message prompts. Brief descriptions of what was captured in the images are presented below.

*Prompt A [“What are the values you hold around this pregnancy?”]*

For prompt A, items pictured in photos included a bench in a wooded area, breakfast food, water cups, and reading a book.

*Prompt B [“What are the challenges you’re facing during this pregnancy?”]*

For prompt B, items pictured in some photos included working from home, a physical activity tracker, a limited personal space corner of the house, and a water bottle representing hydration.

*Prompt C [“What external factors influence your health (positively or negatively) during this pregnancy?”]*

Items pictured for prompt C included the nesting process of setting up a nursery, prenatal vitamins, pets, and family activities.

*Prompt D [“What resources do you have enough of, and what resources do you need more of to support a healthy pregnancy?”]*

Some depictions in the photos received in response to this prompt represented fitness equipment, time spent outdoors, and pregnancy pillows.

*Prompt E [“How are you feeling during this pregnancy?”]*

Items pictured for prompt E included self-care practices, exhaustion, engagement with family, and convenient food options.

*Prompt F: [“How has the COVID-19 pandemic impacted your pregnancy experience?”]*

Items photographed for prompt F included depictions of mask-wearing across different settings, hospital bracelets, and vacation experiences.

*Prompt G: [“What are you doing to take care of yourself during this pregnancy?”]*

For prompt G, some images shared highlighted time spent in nature and healthy behavioral choices (e.g., food prep).

#### Qualitative themes

The main themes identified included challenges related to COVID-19, social support from family, provider, or community, and self-advocacy. Because these interviews captured pregnancy experiences during a pandemic, participants discussed mental health coping mechanisms and how they navigated the health care system while pregnant as a Black/AA person. A summary of the codebook application with selected illustrative quotes is provided in Table 1.

**Table 1:** Participant quotations from interviews organized by theme

Themes	Codes	Illustrative quotes
Pandemic-Related Challenges	Work Changes	*“…So it's been, you know, over a year now [working from home]. So, I'm still trying to get adjusted, which is crazy. Cause it's been so long, but I'm still trying to, trying to adapt to it. I love it. Don't get me wrong…it just seems like it's more now because you know, I'm coming downstairs in my pajamas and you know, I'm not really as focused as I would like to be, which is affecting, you know, my job essentially. So yeah. That's why work stresses me out right now.”*
	Safety Precautions (vaccine, masks, social distancing)	*“…I'm looking around like, why aren’t people wearing that mask? So it's like, I'm trying to have fun, but that's all I'm thinking about. …So it’s bittersweet … I want to start going out … Cause, you know, the world is opening back up, but a spike, bam, pregnant and being out right now. It's not working for me.”*
	Social Isolation	“…*the isolation, you know. I've not hugged my mother and my dad, my niece, my nephews, my brother in over a year. I mean, my family takes COVID very seriously… So the few times I see them, everyone's wearing a mask. Thanksgiving everyone was wearing a mask. We were all eating in separate parts of the house because you can't eat obviously with a mask on,…it's just different. I often think what would my pregnancy have been like if it were without COVID. Having my husband to be able to come to appointments.”*
	Mental Health	*“… I'm from Maryland…so we went up there to …just get away from, COVID, away from the stress of COVID, and of course, we took proper precautions and everything, but still like, how has COVID impacted because* *it's been a lonely ride, this ride has been so freaking lonely. Um, you know, because when you're pregnant, your family is able to celebrate with you. They're able to join in on different visits, on all kinds of things. But, with all the COVID protocols, it's made it a very lonely, depressing journey.”*
	Hospital Restrictions	*“…typically my parents would be there. My brother and sister-in-law would be waiting after the baby's born and you know, that's not possible, so it's definitely a different kind of experience.”*
	Vaccine Hesitancy	*“Well, when they first had the vaccines, I was like, ‘oh no, nope, nope, not me.’ So it never even crossed my mind, but just because I've known a lot of people that know people that have died and they've gotten really, really sick. I was just talking to my doctor and asked her what she thought. And she was like, absolutely a hundred percent I recommend it, but I [was] nervous cause there's not, I just feel like it hasn't been out long enough to know what it's going to do to unborn…I just feel like it personally hadn't been out long enough for me to even get it.”*
Coping: Social Support	Family & Friends	*“I would say my mom had talked to her almost, oh, used to be every day, but now it's, I've been so busy that it's been less and less…probably like every other day I speak with my mother-in-law, believe it or not, a lot. I speak with my husband's grandmother, too; she’s 90. I chat with her a lot on the phone. And…there is a couple of other friends that I have here in Charlotte that I try to keep up with as well as some church brothers and sisters that I have to talk to.”*
	Church Community	*“…In church Sunday, the sermon, um, the pastor was talking about purpose being inside of us all… And I thought about that figuratively, but also literally, that I have a baby inside of me, and there is purpose for her life. I can't wait to see what she does and the impact that she will have on me, my husband, but just on anyone that she encounters…”*
	Healthcare Providers	*“And, and my previous appointment with my OB before this appointment, she really did have, like, um, a tough talk with me. Not, not in a negative way, but a necessary way, because she was just like, I'm starting to get worried about you because you are worried about everything like beyond what you really should. And, you know, she was just like, that's not good for you. That's not good for the baby. Like you need to enjoy your pregnancy.”*
	Online Community	*“The ‘what to expect’ [mobile] app. I live in this little community tab here. There were just so many posts about insomnia and can't sleep, and it's like, [an app member] said, it's ruining my life. And I'm like, I'm right there with you. I know what that's like. …So, it was just me offering any advice at 1:38 in the morning. Hey, you're not alone… Lots of posts like that.”*
Prenatal Health Navigation	Monitoring Health	*“Even with my doctor whom I've known, whom I've trusted, you know, I'm sitting here telling her, okay, well, remember I had this time last pregnancy, so let's check on this, this, okay. But I went one time, and they weren't even to check my blood pressure. If someone had a history of high blood pressure wanting to [receive] a blood pressure. …It just seems like nowadays these appointments are just rushed, and they cause, you know, they give you like 15 min, 20 min. And if you have other concerns, it's like, if you didn't call in with those concerns, you have to set another appointment, or you have to wait. So it was just like, and I don't know if everybody's going through that. I can only go by my own experience, but yeah. That's how I feel.”*
	Health Literacy	“*I think one thing that I have working to my advantage is I am an educated person, and I am not afraid to be articulate about things that I see are problems, but that's not the case for all Black women. And that's not the case. If you don't know to, take notes about questions you have or, you know, to speak up. And I was in one of the groups on the, What to Expect App. And they were like, you know, my doctor just made me feel so stupid, or my doctor just wouldn't answer any of my questions, and I just feel so sad, and I just came home and cried, and I'm not looking forward to my next appointment*.”
	Questioning	*“…there's a 20-min slot for this appointment. I'm taking the full 20 min because there's always things that I want to ask*.”
	Advocate	*“…I think we all have a responsibility to try to help one another, even in the example of my cousin sending me that book, but of course, she sent me other resources… to help, to think about things that we are more likely to encounter in pregnancy, not due to racism, just health issues, different things like that. So you need to be active. You need to be doing this; you need to be doing that. So I just think, um, education and, you know, making sure that we're all doing our part.”*
	Representation in the Healthcare System	*“…My doctor, within the Charlotte network, is a brown lady. She's Indian. So yeah, so like I said, I want to support all brown colors. If that's Black, Indian, you know, whatever Hispanic, uh, not saying that I don't support the white community, but I feel like we need a bigger push than they do.”*

### Pandemic-related challenges

**Fig. 3:**
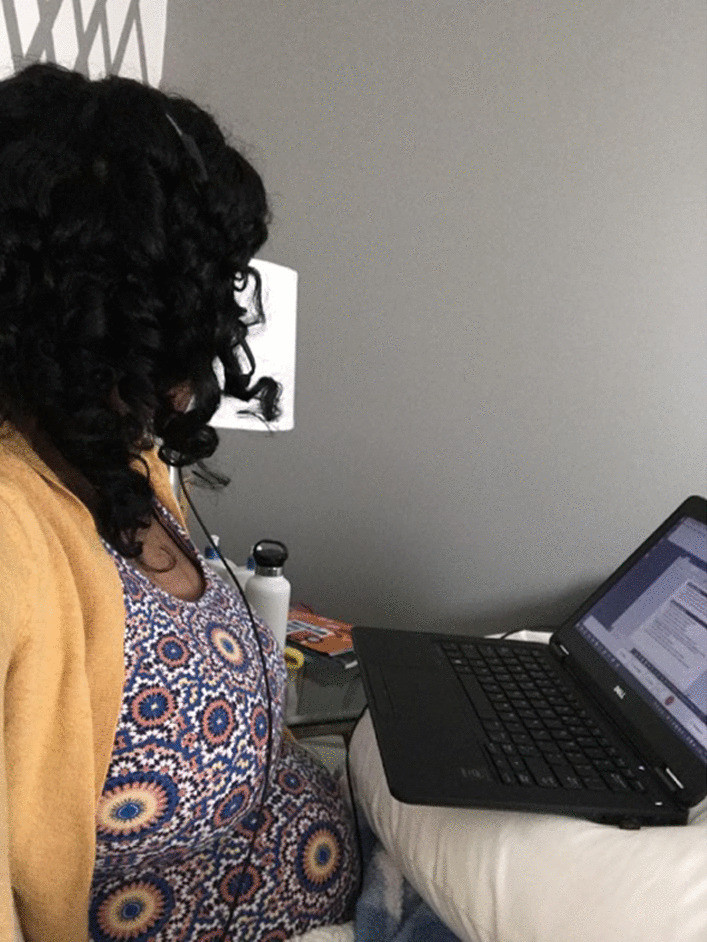
Representative photograph of pandemic-related challenges featuring a participant working from home while pregnant

Of the pandemic-related challenges reported, all participants mentioned safety precautions (i.e., vaccines, social distancing, or wearing masks) that affected their daily activities and their ability to have their support persons present at visits/delivery. One participant reported that while working from home (see Fig. 3) had its pros, the lack of boundaries was negatively impacting her work experience and creating additional stress:* “I’m coming downstairs in my pajamas and you know, I'm not really as focused as I would like to be, which is affecting, you know, my job essentially. So yeah. That's why work stresses me out right now.”*

All participants reported that safety precautions associated with COVID-19 created additional stress during their pregnancy experience. Specifically, participants expressed concerns regarding whether they would contract COVID-19 and pass it to the baby, whether the vaccine was safe to get, and whether the necessary support persons could be in their visits and delivery. One participant noted that while their provider unequivocally recommended the vaccine, she still felt conflicted: “*I just feel like it [the vaccine] hasn't been out long enough to know what it's going to do to [the] unborn.*” Another participant reported concerns about going out in public: *“I'm like looking around like, why aren’t people wearing that mask? So, it's like, I'm trying to have fun, but that's all I'm thinking about.”*

Safety concerns such as these were negatively impacting participants’ mental health, with some reporting awareness of negative psychological changes.

*“I'm too worried about who has Coronavirus, you know, who's going to give it to me. Am I going to get sick? So, I feel I'm pregnant during this pandemic, in general, has made me crazy. I mean, I don't know if I would have been as crazy not being pregnant. It's just that I know pregnancy was like a high risk, so they made me a little bit more crazy.”*

The pregnancy experiences of participants were also influenced by hospital mandates that limited significant others being present for visits/delivery. In one instance, a participant expressed frustration with a rhetorical question: “*I have to pay up to almost a thousand dollars for [either my husband or my doula] to be there with me, to support me through my pregnancy?* “Another participant also reflected on the impact of her significant other being restricted from attending visits: *“I often think what would my pregnancy have been like if it were without COVID, you know…having my husband to be able to come to appointments, you know, at first he could come and then [the hospital system] kind of cracked down.”*

#### Social support

Some participants mentioned that although they had not seen most of their family members and friends in-person as often due to the social distancing measures, there was an increase in communication via phone calls, group chats, and video calls. One participant shared:

*“...probably like every other day I speak with my mother-in-law, believe it or not, a lot. I speak with my husband's grandmother a lot too. She's 90. I chat with her a lot on the phone. And, um, there are a couple of other friends that I have here in Charlotte that I try to keep up with, as well as some church brothers and sisters that I have to talk to.”*

**Fig. 4:**
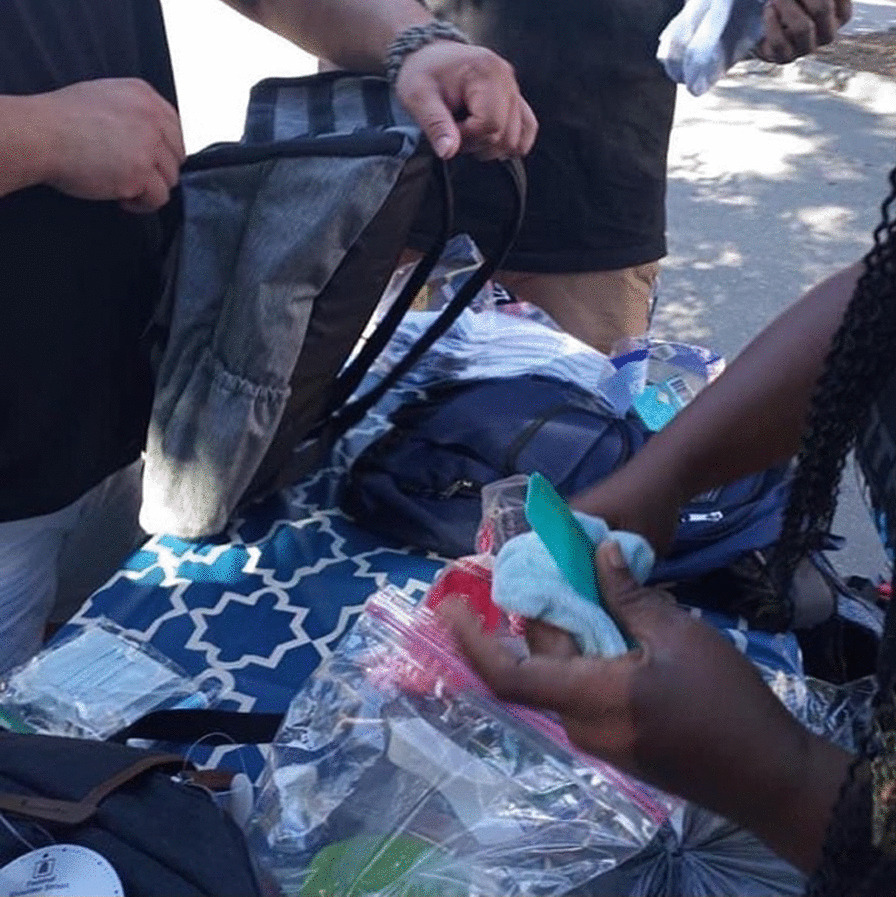
Representative photograph of social support of a participant engaging in community service with her family

Other participants reported finding ways to engage in family or community activities during the pandemic with social distancing in mind (see Fig. 4). For example, when discussing physical activity outdoors, one participant said: *“It gives me energy; it gets us out of the house to enjoy the air outside… It's me and my mom and my aunt. So, it was like family time as well. And then, you know, they got the music. So, to me, it's like my little club.”* One participant took the pandemic as a teachable moment for her family and engaged them in community service to support their neighbors.

Another participant mentioned that her church gave her access to a social support structure and socializing activities. When asked about whom she counted on for social support, the participant shared the following:

*“All I can think of is family again, but not necessarily blood family. I have a group of girls and guys from church that we were part of…but it's mixed…most of us are [of] Black/AA descent. There's one that's Spanish, but just having that camaraderie, like a group of friends that you can act as family with and have dinner prep night and you just kind of eat and laugh and just being the support for one another, you know, just having each other's backs…probably one thing that comes to mind just having that partnership and having support, so and strengthen the community with that as well. I mean, if we're happy, we're happy; we can help the community be happy as well.”*

Additionally, participants mentioned that they also received support from the broader community, whether online via pregnancy apps or in-person through non-profit organizations. One participant, for example, mentioned finding and offering support on an online platform during the night when she was experiencing insomnia: “*The ‘what to expect’ [mobile] app. I live in this little community tab here. There were just so many posts about insomnia and can't sleep, and it's like, [an app member] said, it's ruining my life. And I'm like, I'm right there with you. I know what that's like. …So, it was just me offering any advice at 1:38 in the morning. Hey, you're not alone… Lots of posts like that.”*

Regarding healthcare provider support, participants reported both positive and negative experiences. After having to switch providers, one participant noted a positive relationship:

“*We'll see. She was an African-American doctor… I just liked it. It was a small practice. So, it was three women there. I knew all three of them. I met all three of them, and they just, I just, it's just something about, it just felt good. I mean, my friend had her baby with her, and her daughter is now 18 years old. So, it's like we talk about everything. I just feel like she [the provider] cares about me. You know, my body concerns. Like if something's not right, I feel like she, you know, wants to figure it out.”*

All participants mentioned hospital and stigma consciousness due to race or socioeconomic status. Notably, a participant shared: *"And I just hear about so many people who are like, oh, the hospitals didn't care about them because they're Black, and it's something I don't want to believe…but you hear about it so much. Sometimes I'm like, okay, is it true? And so that's one thing that I worry about sometimes, like, you know, is there a risk of me not coming home after I'm going to give birth in the hospital?".*

Insurance status was mentioned by two participants during the interviews as a barrier to receiving quality health care and a reason for switching providers during the prenatal experience:

*“So, you don't have that good insurance or don't have insurance at all. I mean, if you don't have insurance, they really are not going to care about you. So, um, I think just that portion of it is, is very hard.”*

#### Prenatal health navigation

**Fig. 5:**
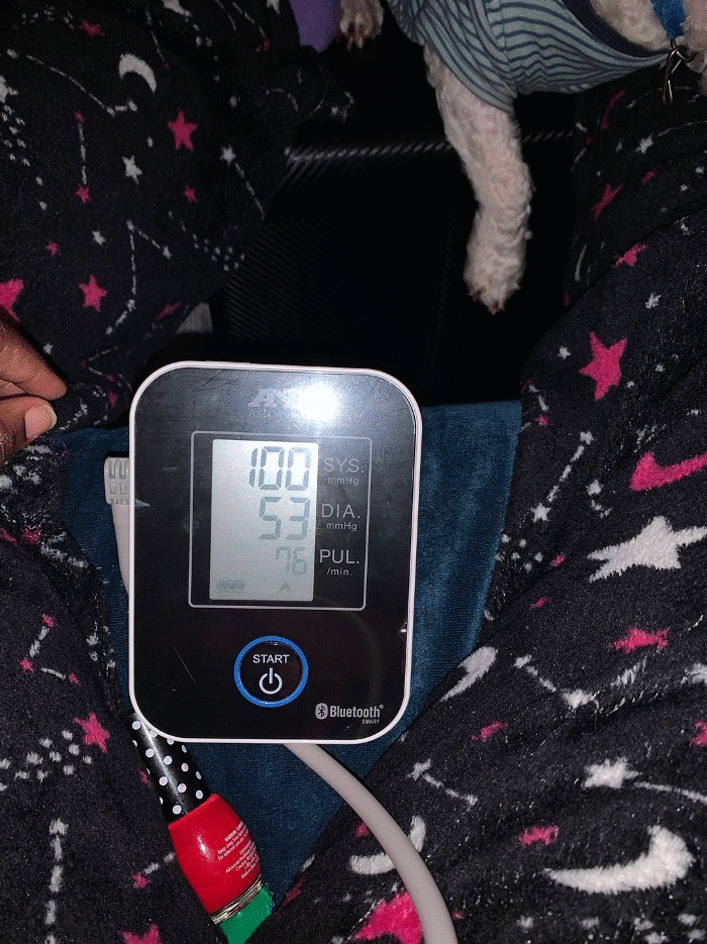
Representative photograph of prenatal health navigation of a participant monitoring her blood pressure from home

Another emergent theme was prenatal health navigation, where participants described their strategies to ensure a healthy pregnancy as a Black/AA birthing person. An underlying expression of this theme was self-advocacy in OB/GYN care. Participants described self-advocacy with an emphasis on necessity due to provider neglect. Three participants explained that they had the experience of having to remind their healthcare providers repeatedly of preexisting health conditions, to self-advocate for tests to be done, or to monitor their symptoms independently:

“*I'm bringing it up, you know, at appointments… don't forget to check my blood pressure, like have my blood sugars [checked], and they're like, well, we don't do that until a certain point. And I'm like, well, I don't want to go through the same thing as I did last time, so we need to stay on it. So, I think the more that I pushed, they gave me the test that I needed.” *(See Fig. 5).

Additionally, participants found it necessary to be prepared for prenatal appointments. Participants noted that if they developed a list of questions and concerns between their appointments to ask their providers, it impacted their healthcare quality:* “Things that I've asked that if I hadn't asked, I don't think I would have gotten that information. And so, I think it's a reminder to be your own advocate.”*

One participant discussed being at an advantage by having a higher education level than others in her community: *“I think one thing that I have working to my advantage is I am an educated person, and I am not afraid to be articulate about things that I see are problems, but that's not the case for all Black women.”*

When asked what participants thought would help improve the Black/AA pregnancy experiences, barriers within the healthcare system were mentioned:

*“I think it should be focused more on, um, just help, of course, you know, making regular appointments, making it easy to get appointments, easy access to help maybe, um, chatting just you have like questions that like this, cause it's hard to go to the doctor all the time, and they're always booked and then the urgent care you spend a $45, you know? So, I think having availability is good. Like I called my doctor [when] I was having some issues, and they were like, oh, we can see you in two weeks. I could be dead in two weeks. You know what I mean? So, I think being there, being available, checking up, asking questions…more than four weeks is just a long time. So, if you're having issues with blood sugar, issues with high blood pressure, you know, it seems like you can kind of be seen regularly so you can get those things checked up on before it's too late.”*

## Conclusions

This study had two primary objectives. First, to describe the experiences of pregnancy and social support among Black/AA women in one Southeastern United States county during the COVID-19 pandemic. Second, to identify the participants’ strategies for coping during their pregnancy experience. We approached this study with a mixed methodology to understand the perceived barriers and strengths present among this population within the context of the COVID-19 pandemic. Our study demonstrated that Black/AA pregnant women in Charlotte, NC, have utilized various strategies to handle the challenges of pregnancy during the global pandemic, emphasizing social support structures and active prenatal health navigation (e.g., self-advocacy).

Our survey findings demonstrated that participants experienced multiple concurrent stressors during the COVID-19 pandemic, with financial, health, and impact on others as the most cited concerns. Economic concerns were most commonly noted, and given the expenses associated with preparing for a baby, medical systems might consider waiving unnecessary fees like late appointment cancellations during a pandemic. Healthcare providers should use OB/GYN visits as a resource checkpoint for supporting Medicaid enrollment to eligible patients and other programs or resources that might alleviate the financial burden during this challenging time. Work-related challenges were described across the survey and qualitative interviews. Working from home provided comfort regarding COVID-19 health exposures but led to more sedentary behaviors and difficulty focusing on tasks.

Participants described safety as a priority but noted conflicts with longing for social support through this life phase. From the qualitative analysis, vaccine hesitancy and safety precautions to prevent COVID-19 showed up for the pregnant person but created additional complexities around planning visits with family and friends. We also learned more about the gravity of giving birth when hospital restrictions limited the amount and types of social support allowed. This combination may lead to social isolation and mental health challenges at a time when social distancing is promoted as an ideal preventative measure for slowing community transmission. There is an opportunity for creating virtual support networks for Black/AA pregnant people to connect during a pandemic.

Understanding the pregnancy experiences of Black/AA women is critical for self-empowerment by centering the voices of women historically excluded from telling their narratives and for racial health equity. Notably, within the context of the COVID-19 pandemic, exploring the sources of stress and coping mechanisms used by Black/AA pregnant women may inform preventative and responsive interventions during future crises. Regarding the negative health behaviors used in response to stressors experienced during COVID-19, we note that screen time and eating (e.g., snacking) behaviors increased. At the same time, our sample reported positive coping behaviors such as talking with family, brain exercises or listening to music. Additionally, participants were involved in more faith-based practices and continued mediation practices to help them cope with the COVID-19 pandemic. These findings provide important considerations for future interventions among the Black/AA pregnant population in Mecklenburg County, NC.

The findings of this study suggest that many of our participants increased talking with family and friends but engaged in fewer activities with them. Additionally, there was improved communication with their healthcare provider during this period. In one of the qualitative interviews, a participant described how she became more involved with community service events and involved her children in these activities. Another participant described using social support to cope and engage in health-promoting behaviors, such as outdoor physical activity classes with her mother. Increasing social support has been demonstrated to improve health outcomes among this population [46]. In a recent study investigating stress and coping among Black/AA pregnant women before and during the pandemic, social support from family and friends, even if not nearby, reduced worry and discomfort among this population [26]. These studies demonstrate the importance of social support from various context-dependent sources during pregnancy for Black/AA pregnant women.

Several women in our study highlighted the usage of resources and social support networks that have been key in navigating the current healthcare system. Many factors contribute to pregnancy health inequities, such as structural racism, barriers to high-quality care, and discrimination by providers. Given the structural nature of this problem, key scholars and advocates call for systemic solutions instead of placing the onus on the individual [3]. Groups like the Black Maternal Health Caucus advocate for systemic change to promote Black/AA maternal health, such as the Black Maternal Momnibus Act of 2021 and the Build Back Better Act of 2021 [47]. The proposed legislation includes the need for culturally humble care, funding to diversify the perinatal workforce, evidence-based interventions, providing respect to midwives and doulas, and standardized practices for the health outcomes of Black/AA mothers [47]. The experiences described by the participants of this study align with the focus of these legislative actions, such as an expansion of Medicaid in North Carolina.

Although policy efforts would be a critical step in the right direction for Black/AA maternal health, policy change can be a lengthy process. There is a mental health cost even for the women who do not die from this structurally sanctioned negligence [26]. The onus should not be on an individual to compensate for a broken system, yet our findings demonstrate that Black/AA women felt the need to advocate for their pregnancy health without structural change; they saw this as a matter of self-preservation. Participants noted that their health literacy, social support, and self-advocacy were strengths while navigating their pregnancy during the COVID-19 pandemic. Implications of these findings include public health recommendations to improve health literacy, patient navigation, and community mental health for Black/AA pregnant women in Mecklenburg County, NC.

In the U.S., the medical field is dominated by White, non-Hispanic Americans [48]. We found that participants preferred being seen by same-race healthcare providers, which aligns with the current literature [49]. Research has suggested that individuals identifying as Black/AA show higher provider distrust than patients who identify as White [50], which is rooted in historically receiving lower-quality care [17, 51]. Hall et al. found that providers had an implicit bias against non-White individuals [51]. These biases translated into negative interactions with the patients, decision-making about treatment, adherence to treatment, and overall health outcomes for the patient. Our study corroborated these findings in Black/AA women in Mecklenburg County, NC; participants reported numerous experiences where they felt unheard by White providers and did not receive the high-quality treatment they expected. Bogdan-Lovis et al. found that while many participants in their study preferred a provider of the same race as themselves, other factors held high value, such as respect, competence, and trust in the partnership [49].

An extension of self-advocacy is preparedness and knowing the critical questions to ask to promote one’s health. In response, participants self-advocate through community support, informing and educating themselves, and developing lists of questions before seeing a healthcare provider. Research shows that women have a history of being dismissed in the U.S. medical system [52], and participants frequently stated that their needs were not met sufficiently and that they had to remind the provider about their pre-existing conditions constantly. Further, participants mentioned how time slots for obstetric appointments were limited and that providers had limited minutes to dedicate to the appointment. This restricted availability frustrated participants, who could not get all the information they needed. Strategies reported by our participants to address the limited time with providers included continuously relaying their medical history to their provider, making lists of their questions in advance, and frequently requesting tests to monitor their health (see Table 1).

In light of the social and structural disadvantages Black/AA women face during their pregnancy and postpartum experience, reproductive advocacy groups have been formed to promote equity in obstetrics and gynecology. These groups include the Black Mamas Matter Alliance [53] and Black Women’s Health Imperative [54]. These groups are founded by empowered women who have taken maternal health advocacy into their own hands by influencing policy at a national level by hosting conferences, developing community toolkits, and centering the voices of Black/AA women.

Structural and social change is ultimately required to achieve maternal health equity. In the interim, Black/AA women may benefit from more engaged proximal systems, such as interpersonal interactions and community-based efforts. This concept is supported by research that shows that even when controlling for social determinants of health, racial disparities in maternal health remain [55, 56]. To maximize the impact of these interventions, it is best to turn to the women of the community themselves to ask about their coping experiences, challenges, and enabling factors.

While our sample noted that self-advocacy was an effective way to promote health, self-advocacy requires a certain degree of health literacy that not all women have the same access to. During an interview, one of our participants reported that while she knew what questions to ask, she perceived that other Black/AA women in her community did not have the same level of awareness. Participants in our sample felt it was necessary to have a solid understanding of their health condition to be adequately prepared to communicate their care plan with their healthcare provider, regardless of their educational attainment. While health systems and government structures take time to develop strategies and implement programs and legislation to address the maternal health crisis, Black/AA women continue to carry the burden of self-advocacy. Becoming educated on maternal health cannot start and stop with the healthcare provider. There is a promising opportunity to support pregnant people by increasing their health literacy through personal research, the support of online communities [57], and health education programs specifically designed to improve maternal health literacy [58]. As maternal health literacy increases, more empowered conversations with healthcare providers can take place, which has the potential to result in more patient-centered care and allows the woman to be the initiator of informed decision-making regarding her health [59].

There are notable strengths and limitations of this study. This project used a mixed-methods approach to empower Black/AA women to share their viewpoints on the pregnancy experience. The use of PEI was a significant strength of this study as this qualitative approach centers the voices of the community by allowing participants to share personal stories rather than the researcher leading the narrative on behalf of the participant [35]. In addition, the interviews contextualized the survey data collected during the pandemic.

The primary limitation of this study was the generalizability of our findings due to the small sample size and narrow geographic focus. Our recruitment and data collection procedures occurred amidst the COVID-19 pandemic (February 2020 to May 2021), making identifying and retaining participants difficult. A disadvantage of the small sample size is the degree to which this qualitative data is representative and thus generalizable to other Black/AA women experiencing pregnancy in the Southeast. Though the sample was small, we quickly reached saturation given the context of the pandemic and social restrictions in place limiting the breadth and depth of daily life experiences. A systematic review of qualitative studies by Hennink and Kaiser (2022) concluded that studies could reach saturation with a small sample when homogenous with narrowly defined objectives [45]. Thus, we primarily focused on using these interviews to contextualize and confirm the survey data collected from a larger sample.

During the COVID-19 pandemic, Black/AA pregnant in Charlotte, NC, faced financial, health, and social challenges. Our findings demonstrate the need for targeted public health efforts to build community skills around self-advocacy for Black/AA women experiencing pregnancy. Such actions may succeed more when they incorporate the strengths in the community, like faith-based practices or social support networks. Regardless of the pandemic context, our study results indicate that self-advocacy may be a critical and protective factor for Black/AA women navigating pregnancy in a broken healthcare system. Notable efforts, such as teaching physicians how to provide culturally competent and patient-centered care, are being made to eradicate racism. Similarly, policies on healthcare delivery can be repositioned to reduce adverse maternal and child health outcomes, but structural changes take time. Meanwhile, there is an opportunity for public health or advocacy groups to intervene at the community level with health literacy, health education, and social support efforts.

## Data Availability

The data that support the findings of this study are available from the corresponding author, AD, upon reasonable request.
